# Uncertainty in classification of death from fatal myocardial infarction: A nationwide analysis of regional variation in incidence and diagnostic support

**DOI:** 10.1371/journal.pone.0236322

**Published:** 2020-07-27

**Authors:** Majbritt Tang Svendsen, Henrik Bøggild, Regitze Kuhr Skals, Rikke Nørmark Mortensen, Kristian Kragholm, Steen Møller Hansen, Signe Juel Riddersholm, Gitte Nielsen, Christian Torp-Pedersen

**Affiliations:** 1 Department of Cardiology, North Denmark Regional Hospital, Hjørring, Denmark; 2 Centre for Clinical Research, North Denmark Regional Hospital, Hjørring, Denmark; 3 Unit of Clinical Biostatistics, Aalborg University Hospital, Aalborg, Denmark; 4 Public Health and Epidemiology Group, Department of Health Science and Technology, Aalborg University, Aalborg, Denmark; 5 Department of Cardiology, Aalborg University Hospital, Aalborg, Denmark; 6 Department of Anesthesia and Intensive care, Aalborg University Hospital, Aalborg, Denmark; 7 Department of Clinical Research, Nordsjaellands Hospital, Hilleroed, Denmark; University of Oxford, UNITED KINGDOM

## Abstract

**Aims:**

The usefulness of mortality statistics relies on the validity of death certificate diagnosis. However, diagnosing the causal sequence of conditions leading to death is not simple. We examined diagnostic support for fatal acute myocardial infarction (AMI) and investigated its association with regional variation.

**Methods and results:**

From Danish nationwide registers, we identified the study population (*N* = 3,244,051) of whom 36,669 individuals were recorded with AMI as the underlying cause-of-death between 2002 and 2015. We included clinical diagnoses, procedures, and claimed prescriptions related to atherosclerotic disease to evaluate the level of diagnostic support for fatal AMI in three diagnostic groups (Definite; Plausible; Uncertain). Adjusted mortality rates, rate ratios, and odds ratios were estimated for each AMI category, stratified by hospital region using multivariable regression models. More than one-third (*N* = 12,827, 35%) of deaths reported as fatal AMI had uncertain diagnostic support. The largest regional variation in AMI mortality rate ratios, varying from 1.16 (95%CI:1.02;1.31) to 1.62 (95%CI:1.43;1.83), was found among cases with uncertain diagnostic supportive data. Substantial inter-regional differences in the degree to which death occurs outside hospital [OR: 1.01 (95%CI:0.92;1.12) - 1.49 (95%CI:1.36;1.63)] and general practitioners determining the cause-of-death at home were present. Minor regional differences [OR: 0.96 (95%CI:0.85;1.07) - 1.16 (95%CI:1.04;1.29)] in in-hospital AMI mortality were observed.

**Conclusion:**

There is significant regional variation associated with recording AMI as a cause-of-death. This variation is predominately based on death certificate diagnoses without diagnostic supportive evidence. Studies of fatal AMI should include a stratification on supportive evidence of the diagnosis.

## Introduction

Cause-of-death statistics derived from death certificates are important for monitoring epidemiologic patterns and developing new healthcare strategies. However, critically, the usefulness of mortality statistics depends on their quality, which varies [1–3]. To ensure valid comparisons within and between countries, clear and detailed diagnostic classification groups have been developed in the International Classification of Diseases (ICD) [4]. Nevertheless, substantial regional variation in death from ischemic heart disease (IHD) is present [5–8]. Whether low validity of the cause-of-death statements in death certificates is responsible for the variation remains unexplored.

Mortality statistics rely on the coding done by the individual physicians in which the underlying cause-of-death is estimated with some degree of uncertainty when an autopsy is not performed. Physicians are responsible for stating which morbid condition led directly to death and indicating any preceding conditions giving rise to this cause [9]. Yet, in everyday practice, diagnosing the causal sequence of conditions leading to death is not simple. The data available for the physicians producing the death certificates may vary markedly. The physician may have a hospital file with multiple diagnostic information or, at the other extreme, the physician may have hardly any information. Coding practices in the latter case could, in principle, markedly influence the mortality rate of reported acute myocardial infarction (AMI) and other diseases that physicians are inclined to diagnose when there is little clinical information available.

Misclassification with respect to death certificate completion has been found to be very high, ranging from 25% to 78% in hospital-based studies [10–13], and from 16% to 56% in population-based studies [14–16]. Age, speciality, training, and skills of certifiers are important predictors of misclassification [16]. The influence on mortality statistics has been observed to be particularly high in countries where residents predominantly die outside the hospital and where the autopsy rate is low [17]. In Denmark, at present, the autopsy rate is below 10% [9, 18]. A relatively high sensitivity of AMI in Danish mortality statistics compared to clinical records has previously been reported [19]. Yet international studies have expressed concern about the quality of data from death certificates for analysing mortality related to IHD [20]. Coronary heart disease appears to be over-diagnosed. In cases of sudden death it was found that the reported cause-of-death had to be changed in 30% of cases after an autopsy was performed, and in 50% of cases where the underlying cause-of-death was reported as IHD, there was no basis for this diagnosis [17].

Regional variation in mortality from AMI could be due to genetic factors, lifestyle, and sociocultural heterogeneity, but could also reflect variability in coding practice. To address this issue, we examined the prevalence of low diagnostic support for fatal AMI in a nationwide sample of 3,244,051 Danish residents and studied whether cases with uncertain diagnostic supportive data impact upon regional variability in incidence in mortality from AMI. Time trends in AMI mortality with respect to diagnostic support and certifier practice were further addressed.

## Methods

### Data sources

In Denmark, all residents are registered with a personal and permanent civil registration number that enables individual-level linkage of nationwide administrative registries. Since the majority of all hospital- and general-practitioner-based medical care in Denmark is reimbursed by the national health authorities, these administrative registries enable accurate population-based studies with national coverage and high levels of completeness [21]. Information on date and place of death, autopsy, and the cause-of-death classified according to the ICD was obtained from the Danish Registry of Causes of Death [9]. From the National Patient Registry we obtained data from patient records including information on all somatic admissions, procedure codes, and discharge diagnoses classified according to the 10th revision of the ICD [22]. We obtained information on all prescription medicines dispensed, classified according to the Anatomical Therapeutic Chemical (ATC) classification system, from the Danish National Prescription Registry. Drug expenses are partially reimbursed by the Danish Health Authorities, providing a valid and accurate register [23]. From the Danish National Health Service Register for Primary Care we identified death certificate registrations completed by general practitioners using reimbursement for the registration disbursed to the general practitioners [24]. Date of birth, vital status and official residence were obtained from the Central Personal Registry [25].

### Study population and study variables

In this nationwide, register-based cohort study, the study population comprised the total Danish population (*N* = 3,244,051) aged 35 years or older registered in the Civil Registration System January 1, 2002. From the study population we identified individuals recorded with AMI (ICD-10: I21-I22) as the underlying cause-of-death (*N* = 36,667) within the observation period from 1 January 2002 to 31 December 2015. To assess diagnostic support for fatal AMI we examined these deaths using individually based comparison of death certificates with hospitalisations and discharge diagnoses, surgery, outpatient procedures, and prescriptions related to IHD.

Discharge diagnoses comprised all ischemic-related heart diseases (ICD-10: I20-I25) including among others angina pectoris, acute coronary thrombosis, and chronic IHD. Furthermore, coronary-artery-related surgical procedures (Procedure code: KNF) such as coronary artery bypass grafting and percutaneous coronary intervention were used as proxies for former ischemic-related heart disease. Hospitalised patients only recorded with suspicion of AMI were likewise identified (ICD: Z034). The medication was restricted to comprise pharmaceuticals prescribed on the indication of acute IHD, namely Nitrates (ATC: C01DA02) and dual antiplatelet therapy (DAPT). DAPT was defined as concomitant use of low-dose acetyl salicylic acid, Aspirin (ATC: B01AC06), and a P2Y_12_ antagonist. Currently, three P2Y_12_ antagonists are available in Denmark: Clopidogrel (ATC: B01AC04), Prasugrel (ATC: B01AC22), and Tricagrelor (ATC: B01AC24). Therapy use was defined as having filled prescriptions within 180 days prior to death.

### Diagnostic support for fatal AMI

Based on the extent of diagnostic support for fatal AMI, the study population was allocated in one of three diagnostic AMI groups: Definite; Plausible; Uncertain. Patients who underwent autopsy or were formerly diagnosed with AMI (ICD-10: I21-I22), who died within 30 days from AMI hospitalisation were categorised as Definite AMI. Conversely, those who died more than 30 days from AMI hospitalisation, those who claimed prescriptions of Nitrates or DAPT, or were formerly diagnosed with an IHD diagnosis (ICD-10: I20-I25) other than AMI, or had surgery related to IHD, or were diagnosed with only suspicion of AMI (ICD-10: Z034) were categorised as Plausible AMI. All other individuals, without diagnostic support related to IHD, were classified as Uncertain AMI.

### Outcome, exposure, and potential confounders

The primary outcome of interest was the extent of diagnostic support, estimating the risk of AMI death defined with respect to the three diagnostic AMI groups (Definite; Plausible; Uncertain). A secondary outcome included whether the general practitioner reported AMI as the underlying cause-of-death, identified by invoices from primary care institutions (invoice number: 2131 and/or 2135). The main exposures were the five official Danish regions responsible for hospital services (North; Central; South; Zealand; Capital). In each region, dedicated and invasive coronary care units with specialists in cardiology and accessibility to diagnostic methods exist. In Denmark, the healthcare system is organised through three administrative levels: state, region, and local. The state holds the overall responsibility for regulation to ensure high standard of care, evidence-based treatment, and up-to-date treatment in all regions. Regulation takes place through, among other things, national guidelines and national quality monitoring systems [26]. Place of death was handled as a potential confounder and was categorised into death outside hospital (place of death: residential address; known address; unknown address) or death in hospital. We also estimated comorbidity by calculating a 10-year Charlson Comorbidity Index score [27] based on hospital discharge diagnosis codes 10 years before death and antidiabetic medication (ATC: A10) 180 days before death, divided into two groups (low/moderate (score:0–2); high (score:≥3)). Potential confounders also included age at death, sex, and calendar-year.

### Statistics

Categorical variables are presented using percentages and continuous variables using medians with the 25^th^ (Q1) and 75^th^ (Q3) percentiles. Chi-square and Kruskal-Wallis tests were performed to test differences between diagnostic AMI groups. Age-standardised specific mortality rates per 100,000 person years for each of the three diagnostic AMI groups by gender and region were calculated with direct age-standardisation to the European standard population [28]. Corresponding confidence intervals (95%) were calculated assuming a Poisson distribution. Mortality rate ratios for the different Danish regions, adjusted for age, sex, and calendar-year were computed using negative binomial regression analysis. To evaluate trends in specific mortality of the three AMI groups, we estimated the average annual change using linear regression models. Odds ratios describing the risk of Definite, Plausible, and Uncertain AMI, compared to the reference all-cause mortality (exclusive death from AMI) among all deaths in the study population, across regions were estimated using multinomial logistic regression models. Multinomial logistic regression analysis was further used to estimate absolute risks of fatal AMI. We tested for interaction using the likelihood ratio test. Significant interactions were found between region and place of death and, therefore, we stratified our analyses. Odds ratios describing differences in general practitioners reporting AMI as underlying cause-of-death across regions among death from AMI were assessed using logistic regression. A two-sided *P*-value <0.05 was considered statistically significant. Analyses were performed using SAS (version 9.4, SAS Institute, Cary, NC, USA), and R statistical software (version 3.3.2, R Development Core Team) [29].

### Ethics

This study was approved by the Danish Data Protection Agency (2008-58-0025, local reference: 2016–3). According to Danish legislation, application for ethical approval is not required for registry-based studies.

## Results

During the study period from 2002 to 2015, 46,721 citizens aged 35 years or older were recorded in the Danish Registry of Cause-of-death with AMI as the underlying (*N* = 36,667 or 78.5%) or contributory (*N* = 10,052 or 21.5%) cause-of-death. Among the 36,667 citizens recorded with AMI as the underlying cause-of-death, 56.3% occurred in men, 54.2% occurred in hospitals, and the median age at death in the study population was 80.5 [Q1,Q3: 71.3, 87.1]. The most frequently used contributory causes of death in relation to AMI as the underlying cause-of-death were AMI without specification (ICD-10: I219), followed by atherosclerotic heart disease (ICD-10: I251) and atherosclerosis without specification (ICD-10: I701).

### Diagnostic support for fatal AMI

Based on diagnostic supportive data for fatal AMI, the 36,667 citizens recorded with AMI as the underlying cause-of-death were categorised according to diagnostic AMI groups (Definite; Plausible; Uncertain). A total of 36.3% (*N* = 13,317) citizens were categorised as Definite AMI (died within 30 days from AMI hospitalisation), 28.7% (*N* = 10,525) as Plausible AMI (antecedent conditions, procedures or claimed prescriptions related to IHD), whereas 35% (*N* = 12,827) were classified as Uncertain AMI (no diagnostic supportive data related to IHD). AMI mortality stratified by diagnostic support is summarised in Table 1. When AMI as a contributory cause-of-death was included in the analysis, the percentage-wise distribution of AMI categorisation was almost the same. The median age at death (78.5 (Q1, Q3: 67.2, 86.5)) for Uncertain AMI diagnosis was significantly lower compared to other diagnostic AMI groups (*P*< 0.0001). Residents without diagnostic support for AMI also presented with markedly fewer comorbidities and claimed prescriptions (*P*< 0.0001).

**Table 1:** Demographic stratification according to diagnostic support for fatal acute myocardial infarction.

	Definite AMI (n = 13,317)	Plausible AMI (n = 10,525)	Uncertain AMI (n = 12,827)	Total (n = 36,669)	p-value
**Sex**					
Women	6108 (45.9)	4164 (39.6)	5747 (44.8)	16019 (43.7)	
Men	7209 (54.1)	6361 (60.4)	7080 (55.2)	20650 (56.3)	<0.0001
**Median [Q1,Q3] age [years]**	81.4 (73.6,87.1)	81.4 (73.3,87.6)	78.5 (67.2,86.5)	80.5 (71.3,87.1)	<0.0001
**Place of death**					
Outside hospital	921 (6.9)	4751 (45.1)	6541 (51.0)	12213 (33.3)	
Hospital	12138 (91.1)	4097 (38.9)	3645 (28.4)	19880 (54.2)	
Unknown	258 (1.9)	1677 (15.9)	2641 (20.6)	4576 (12.5)	<0.0001
**Comorbidity**					
Stroke	3285 (24.7)	2759 (26.2)	2119 (16.5)	8163 (22.3)	<0.0001
Peripheral artery disease	1970 (14.8)	1724 (16.4)	923 (7.2)	4617 (12.6)	<0.0001
Heart failure	5305 (39.8)	4977 (47.3)	1651 (12.9)	11933 (32.5)	<0.0001
Hypertension	5118 (38.4)	4482 (42.6)	3028 (23.6)	12628 (34.4)	<0.0001
Atrial fibrillation	2881 (21.6)	3333 (31.7)	1770 (13.8)	7984 (21.8)	<0.0001
Diabetes	2655 (19.9)	2127 (20.2)	1261 (9.8)	6043 (16.5)	<0.0001
Cancer	2103 (15.8)	1777 (16.9)	1812 (14.1)	5692 (15.5)	<0.0001
**Medicine**					
Nitrates	3627 (27.2)	4482 (42.6)	0 (0.0)	8109 (22.1)	<0.0001
Beta Blockers	4936 (37.1)	5320 (50.5)	2224 (17.3)	12480 (34.0)	<0.0001
Lipid lowering medications	3729 (28.0)	4316 (41.0)	1582 (12.3)	9627 (26.3)	<0.0001
Aspirins	6800 (51.1)	6977 (66.3)	3450 (26.9)	17227 (47.0)	<0.0001
Antiplatelets	1460 (11.0)	1296 (12.3)	139 (1.1)	2895 (7.9)	<0.0001
Calcium antagonists	3728 (28.0)	2646 (25.1)	2343 (18.3)	8717 (23.8)	<0.0001
ACE-inhibitors	5147 (38.6)	5170 (49.1)	3467 (27.0)	13784 (37.6)	<0.0001
Diuretics	7645 (57.4)	7457 (70.9)	5613 (43.8)	20715 (56.5)	<0.0001
Antidiabetics	2267 (17.0)	1841 (17.5)	1285 (10.0)	5393 (14.7)	<0.0001

Characteristics of residents (*N* = 36,669) aged ≥35 years or older, recorded with acute myocardial infarction (AMI) as the underlying cause-of-death by diagnostic support for fatal AMI. Data are presented as median with interquartile range (age) or number of residents and percentage (all others).

### Regional variation in diagnostic support for fatal AMI

**Fig 1:**
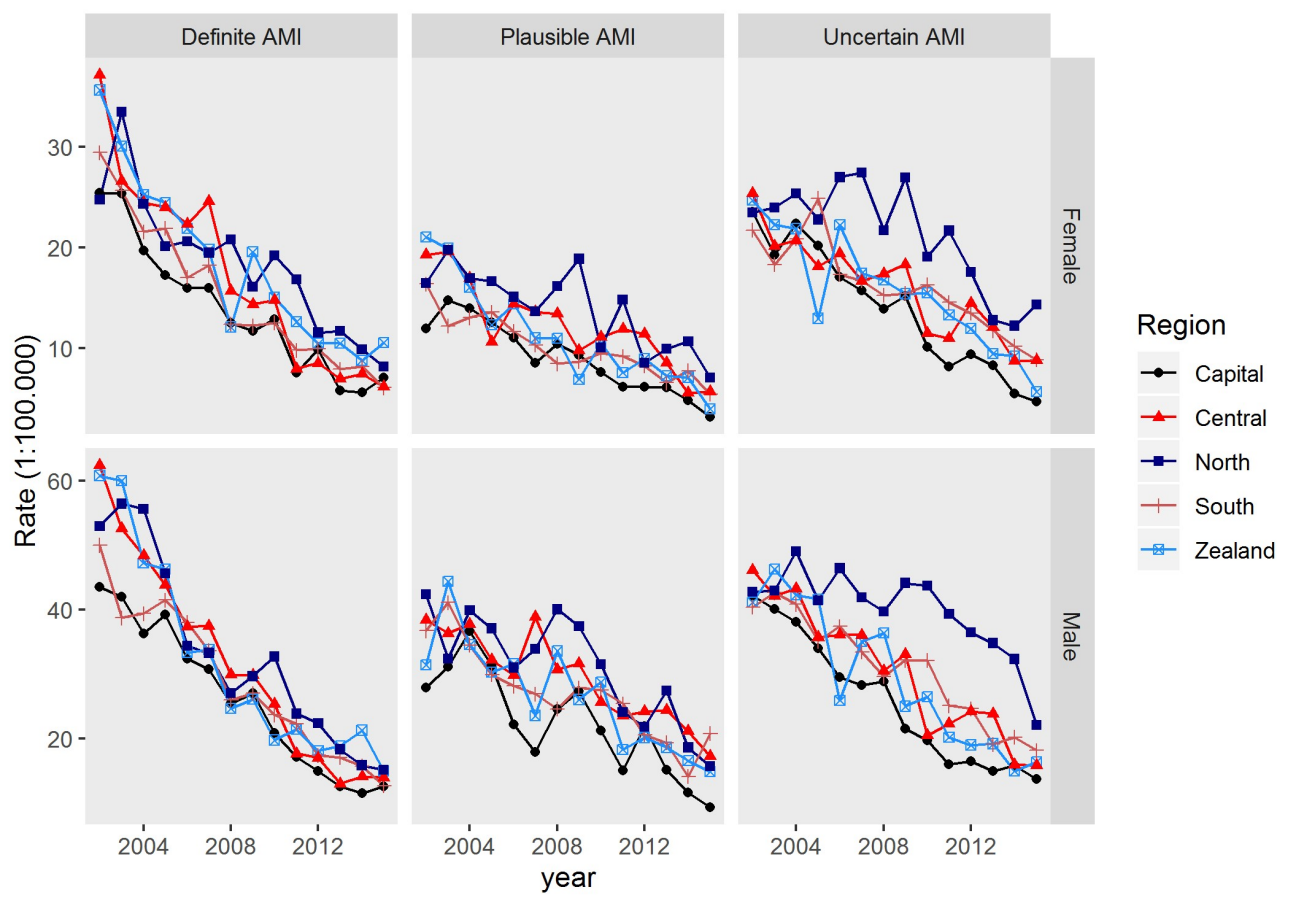
Age-standardised mortality rates from acute myocardial infarction by region and diagnostic support. Age-standardised mortality rates from acute myocardial infarction (AMI) per 100,000 population, stratified by gender and by diagnostic support for fatal AMI, for each official Danish region (*N* = 36,669).

**Fig 2:**
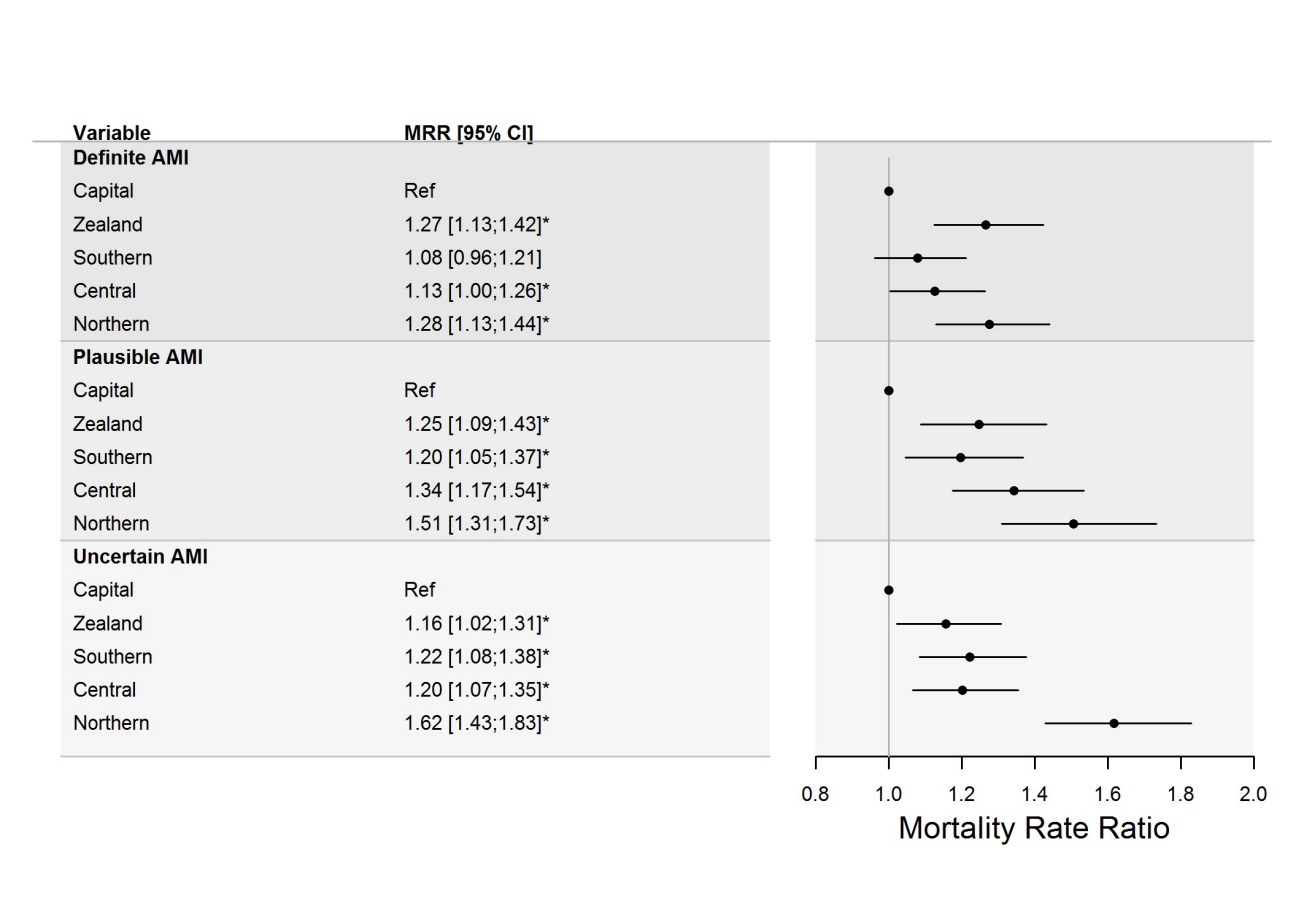
Standardised mortality rate ratios from acute myocardial infarction by diagnostic support and region. Forrest plot presenting standardised mortality rate ratios from acute myocardial infarction (AMI) by diagnostic support for fatal AMI with their 95% confidence intervals for each official Danish region (*N* = 36,669), compared to the Capital Region of Denmark (reference).

Mortality rate ratios (MRR), adjusted for sex, age, and calendar-year showed significant regional variability in total AMI mortality with ratios of regions ranging from 1.16 (95%CI:1.02;1.33) to 1.47 (95%CI:1.29;1.68), compared to the largest region, the Capital Region of Denmark (reference); [Zealand: MRR: 1.22 (95%CI:1.07;1.39)]; [South: MRR: 1.16 (95%CI:1.02;1.33)]; [Central: MRR: 1.22 (95%CI:1.07;1.39)]; [North: MRR: 1.47 (95%CI:1.29;1.68)]). The age-standardised mortality rates of the three diagnostic AMI classification groups, stratified by sex and hospital region, are plotted in Fig 1. Plots with corresponding confidence intervals are available in the data supplement (S1 Fig). The largest regional variation in death from fatal AMI was found among the 12,827 individuals who lacked diagnostic support for fatal AMI (Uncertain AMI) with all regions differing significantly from the reference and MRR of regions varying from 1.16 (95%CI:1.02;1.31) to 1.62 (95%CI:1.43;1.83) compared to the Capital Region of Denmark (reference) (Fig 2). In contrast, the lowest regional variation in fatal AMI cases was observed in residents who died within 30 days from AMI hospitalisation (Definite AMI), with regional MRR ranging from 1.08 (95%CI:0.96;1.21) to 1.28 (95%CI:1.13;1.44), compared to the Capital Region of Denmark (reference) (Fig 2).

### Time trends in AMI mortality

From 2002 to 2015, the age-standardised mortality rate from AMI per 100,000 person years declined in men from 123.4 (95%CI:118.2;128.8) to 45.5 (95%CI:42.7;48.6) and in women from 67.4 (95%CI:64.3;70,5) to 20 (95%CI:18,4;21.7) (Table 2). A substantial decline in mortality rates (annual change) was observed across all three diagnostic AMI classification groups and for both sexes, with the greatest declines in individuals classified as Definite AMI (Table 2). In addition, the total AMI mortality rate fell the most (annual change) among patients who died in hospital compared to residents who died outside the hospital.

**Table 2:** Trends in mortality rates from acute myocardial infarction by gender, diagnostic support, and place of death.

	2002	2015	Overall change (%)	Annual change (%)
**Total AMI mortality**				
Men	123.4	45.6	- 63.0	- 6.56 (95%CI:-7.05; -6.07)
Women	67.4	20.0	-70.3	- 3.70 (95%CI-3.89; —3.52)
**Definite AMI**				
Men	48.4	13.0	- 73.3	- 3.10 (95%CI -3.42; -2.77)
Women	28.7	7.1	- 75.3	- 1.72 (95%CI—1.94; -1.50)
**Plausible AMI**				
Men	34.2	15.5	- 54.7	- 1.48 (95%CI -1.75; -1.22)
Women	15.7	5.0	- 68.2	- 0.81 (95%CI -0.90; -0.73)
**Uncertain AMI**				
Men	40.8	17.0	- 66.1	- 1.98 (95%CI -2.14; -1.82)
Women	23.0	7.8	- 58.3	- 1.17 (95%CI -1.29; -1.04)
**Death in hospital**				
Men	80.7	20.2	-76.3	-5.04 (95%CI -5.60; -4.47)
Women	41.8	9.9	-74.3	-2.63 (95%CI -2.90; -2.33)
**Death outside hospital**				
Men	34.6	17.1	- 50.6	- 1.67 (95%CI -2.09; -1.26)
Women	22.2	7.3	- 67.1	-1.16 (95%CI -1.39; -0.92)

Mortality rates from acute myocardial infarction (AMI) by gender, diagnostic support for fatal AMI, and place of death describing trends per 100,000 population. Overall change (year: 2002–2015) and annual change are presented in percentwise change and with 95% confidence intervals for annual change.

### Regional differences in place of death

**Fig 3:**
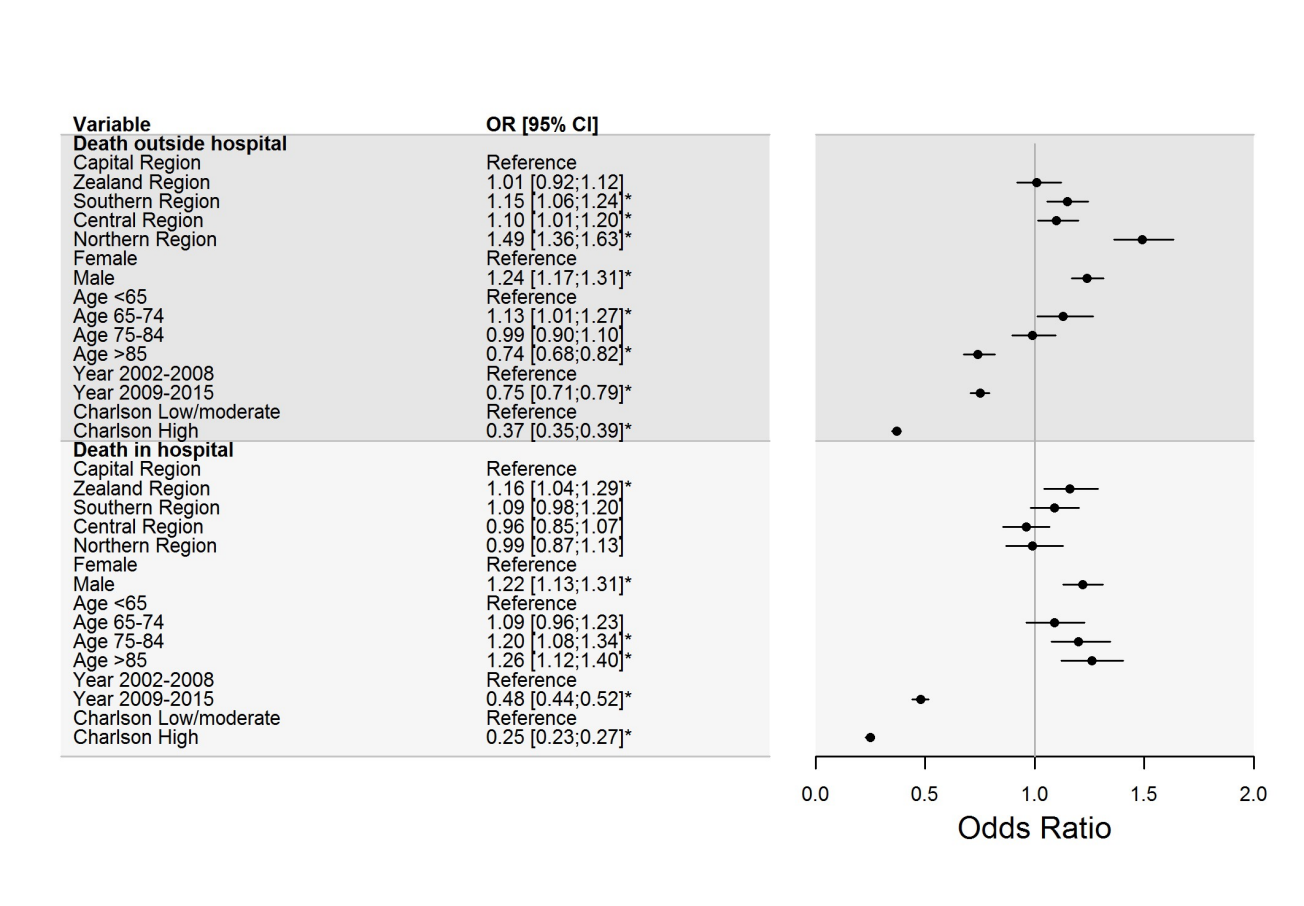
Risk of uncertain acute myocardial infarction across regions by place of death. Multivariable multinomial logistic regression model describing risk (odds ratios), with corresponding 95% confidence intervals, of Uncertain acute myocardial infarction (AMI) compared to all-cause mortality across official Danish regions, stratified by place of death. Model adjusted for covariates. The Reference in the model was the Capital Region of Denmark.

**Fig 4:**
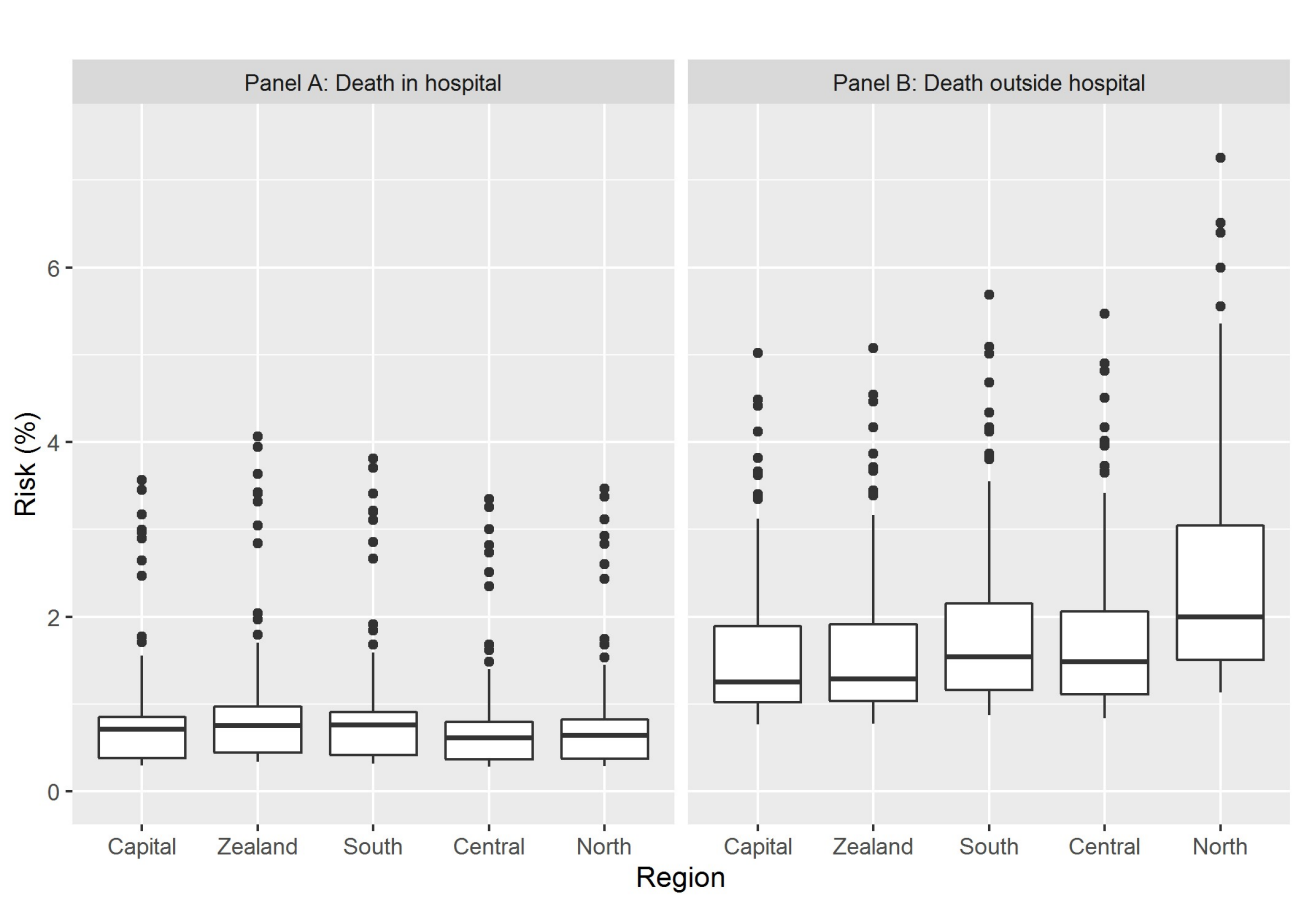
Absolute risk of fatal acute myocardial infarction stratified by region and place of death. Predicted absolute risk of fatal acute myocardial infarction (AMI), stratified by region and by place of death (Panel A: Death in hospital; Panel B: Death outside hospital). Boxplots show 5%, 25%, 50%, 75%, and 95% quantiles of differences in predicted absolute risks.

When place of death was included in the adjusted multinomial regression analysis (confounders: sex, age at death, calendar-year, comorbidity, place of death), estimating risk of AMI death with respect to diagnostic AMI groups, a significant interaction between region and place of death was observed (*P*< 0.0001). Therefore, the analyses were repeated stratified on place of death. The stratified analysis showed significant regional variation in AMI cases without supportive data occurring outside the hospital in three out of four regions, with odds ratios of risk of death from AMI within regions ranging from 1.01 (95%CI:0.92;1.12) to 1.49 (95%CI:1.36;1.63), compared to the Capital Region of Denmark (reference) (Fig 3). Among residents who died in hospital, the variability across regions in individuals without diagnostic support for fatal MI was lower with odds ratios of risk of death from AMI within regions varying from 0.96 (95%CI:0.85;1.07) to 1.16 (95%CI:1.04;1.29) (Fig 3). The predicted absolute risks of death from AMI without supportive data across regions, stratified on place of death, showed higher risk of death from AMI outside hospital and larger variability in death from AMI across regions outside hospital, compared to AMI death without supportive data in hospital (Fig 4).

### Geographic variation in certifier practice

In logistic regression analysis, adjusted for age, sex, and calendar-year, significant inter-regional differences in general practitioners reporting AMI as underlying cause-of-death were observed compared to the Capital Region of Denmark (reference): [Zealand: OR: 1.13 (95%CI:1.05;1.22)]; [South: OR: 1.35 (95%CI:1.26;1.44)]; [Central: OR: 1.55 (95%CI:1.46;1.66); [North: OR: 1.67 (95%CI:1.56;1.80)].

## Discussion

The main result of this nationwide study is that nearly four in ten individuals recorded with AMI as the underlying cause-of-death lacked diagnostic support for fatal AMI. The pronounced regional variability in death from AMI, with nearly 50% higher risk of a fatal outcome of AMI in some regions, is predominantly driven by cases without diagnostic supportive data. An uneven regional distribution of fatal AMI cases without supportive data occurring outside hospital was found, whereas minor regional differences in in-hospital AMI mortality were observed.

A possible explanation for the significant regional differences in fatal AMI outcome is misclassification of AMI as cause-of-death. In support of our findings, a recent study on geographical variation in fatal outcome of AMI demonstrated no geographical inequality in AMI mortality among patients who survived 28 days after AMI diagnosis [7]. The lack of regional differences in AMI incidence rates strongly support our hypothesis of misclassification of AMI as a cause-of-death when limited diagnostic supportive data are present. Regional differences in standard of treatment and procedures including, among other things, accessibility to specialised coronary care units and diagnostic techniques could potentially lead to unequal death from AMI across regions. However, the Danish Society of Cardiology has endorsed international guidelines [30, 31], developed by the European Society of Cardiology (ESC), into the national Danish recommendations to ensure evidence-based and up-to-date treatment throughout the country. These guidelines are widely adapted by Danish cardiologists and the implementation of ESC guidelines are likely to contribute to uniform treatment of AMI patients across regions. In support of equal treatment of AMI patients, Kjærulff *et al*. [7] recently found that no geographical inequality among Danish patients who survived the first 28 days after AMI hospitalisation exist. This particular finding indicates uniform treatment of AMI patients across regions in Denmark, and thereby, that geographical differences in mortality from AMI are less likely to result from unequal accessibility to diagnostic methods and treatment across the country. In addition, regional differences in late accessibility to invasive coronary care units have been examined. Mixed results have been found [32, 33]. However, the most recent study demonstrated that distance to an invasive coronary care unit was not associated with survival in out-of-hospital cardiac arrest patients, but hospitalisation at an invasive coronary care unit improved survival [33].

In line with our findings, indicating uncertainties in the reporting of AMI as cause-of-death, former studies on the validity of the diagnosis of AMI also found that AMI as cause-of-death demonstrated lower accuracy compared to hospitalisation data, with a maximum positive predictive value of 59% [34]

Furthermore, Lowel et al. found that the positive predictive value of AMI was lower for patients who had less clinical data and test results with electrocardiogram and enzyme level available [35]. These results support our findings of more uncertainty in diagnosing and reporting AMI as cause-of-death when limited diagnostic support is present. Contrary to our findings, another Danish study that validated AMI in national registries found relatively high sensitivity compared to clinical records [19]. However, in line with our findings, they also found that the predictive value and the sensitivity were higher for admissions compared to mortality data [19]. Several factors may influence the correctness of death certification. Among these are incidental changes in coding rules and different use of diagnostic criteria, diagnostic techniques, and biomarkers over time [36]. Moreover, age, speciality, training, and skills of certifiers have been found to be important predictors of misclassification [16]. The quality of research may also be seriously impacted since most databases are not established for research. Instead, diagnoses and procedures are submitted by physicians and other healthcare personnel to obtain reimbursement [34].

In the present study, we found substantial inter-regional differences in the proportion of residents who died outside hospital and, furthermore, that death occurring outside hospital was significantly associated with fatal AMI outcome without supportive data. Regional differences in AMI mortality could potentially reflect differences in the degree to which death occurs outside hospital across regions and general practitioners determining the cause-of-death at home without support from diagnostic techniques. Differences in mortality structures have previously been suggested to be partly attributable to differences between rural and urban populations [37]. The population in the Capital Region is entirely urban, whereas the urban population in the North Denmark Region is smaller than the rural population.

There may exist important discontinuity in research projects that rely on the correctness of cause-specific mortality. Previous studies have identified several problems with cause-specific mortality statistics across regions [38]. While some causes of death (e.g. traffic accidents) have somewhat comparable mortality rates across regions, there is a larger degree of geographical difference in the prevalence of other cause-specific diseases. For some causes, the degree of the variation is believed to be too large and therefore researchers have previously claimed that the variation is more likely to be artificial rather than indicative of natural occurence [38]. It is plausible that the regional variation in fatal AMI observed in this present study partly reflects variation in coding practice rather than real inter-regional differences in AMI mortality. Our stratified analysis revealed that when comparing only death outside hospital across regions, significant regional variation in death from AMI was still present. This particular finding indicates possible regional variation in coding practice among physicians recording cause-of-death outside hospital. In everyday clinical situations, determining the causal sequence leading to death is not simple, and, as discussed by some researchers, in a number of cases it will be the most approriate choice for the physician to state that the cause-of-death is unknown, rather than choosing an unfounded cause [39]. However, when a physician currently reports an unknown cause-of-death, this statement will result in more time-consuming administrative procedures involving more institutions. International guidelines for the registration of causes of death [40] instruct that the cause should be recorded as precisely as possible, even if diagnostic support is lacking. Information from the relatives on recent pain in the chest of the deceased without any other information should, therefore, lead to AMI being reported as the cause of death. Our findings of a higher decline (overall change) in in-hospital mortality are consistent with findings from other studies on trends in out-of-hospital deaths due to cardiovascular disease [41]. In case of a sustained decline in in-hospital AMI mortality a larger quantity of death certificates completed without support from diagnostic techniques may be expected prospectively. Inaccurate death certificates impair the precision of national health information data, reducing the utility for public health decision making and research [1].

### Limitations

The strengths of this study included the large sample size, the register-based, and the nationwide design of the study. In this way, we lowered the risk of selection bias attributed to geographic differences in patient characteristics. Furthermore, the retrospective and independent collection of data lowered the risk of information bias. The inherent weakness of the study arises from the indirect character of the method applied. As we used nationwide registries to analyse cause-specific mortality, we can only make indirect assessments of the quality and validity. Extensive inter-regional variation was present; however, we cannot be sure whether this is caused by misclassification as a result of lack of diagnostic support or by real differences in mortality from AMI within the population.

## Conclusion

In conclusion, more than one-third of deaths reported as fatal AMI lacked diagnostic support. Our results strongly indicate that regional differences in AMI mortality are associated with fatal AMI cases without supportive data. This might result from differences in the degree to which death occurs outside hospital and general practitioners determining the cause-of-death at home. These findings emphasise that the use of AMI as a cause-of-death requires attention to supportive data. We suggest that future studies on fatal AMI should include a stratification on supportive evidence of the diagnosis.

## Supporting information

S1 Fig Age-standardised mortality rates from acute myocardial infarction by region and diagnostic support. Age-standardised mortality rates from acute myocardial infarction (AMI) per 100,000 population, with corresponding confidence intervals, stratified by gender and by diagnostic support for fatal AMI, for each official Danish region (*N* = 36,669) between 2002 and 2015.
(TIFF)

## Abbreviations

AMI: Acute myocardial infarction
ICD: International Classification of Diseases
ATC: Anatomical Therapeutic Chemical
IHD: Ischemic heart disease
MRR: Mortality rate ratio
ESC: the European Society of Cardiology
