# Supplementary figures and images for: Uncertainty in classification of death from fatal myocardial infarction: A nationwide analysis of regional variation in incidence and diagnostic support
Source: PLoS One. 2020 Jul 27;15(7):e0236322. doi: 10.1371/journal.pone.0236322 (PMC7384617; doi:10.1371/journal.pone.0236322)

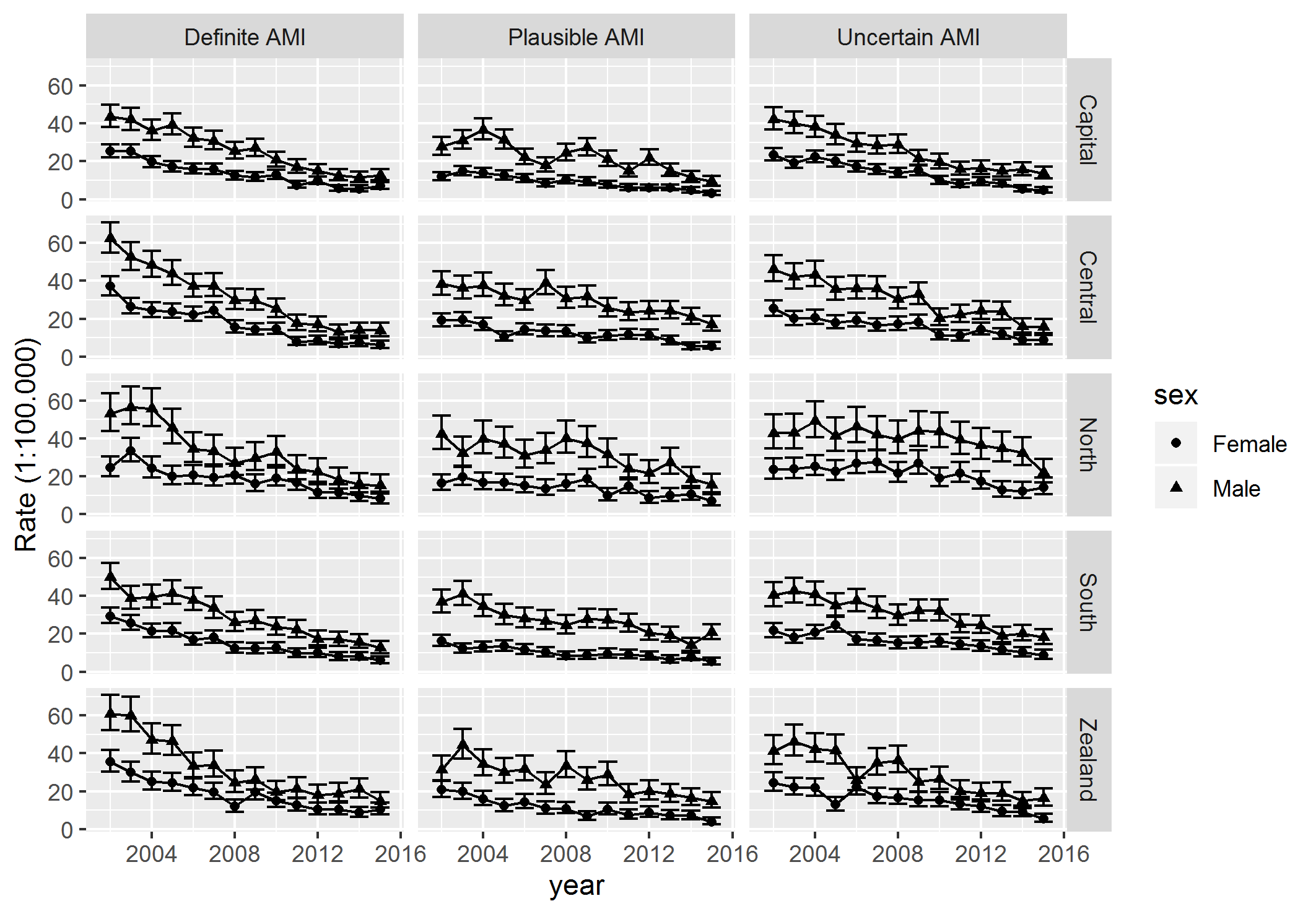

Supplement: S1 Fig — Age-standardised mortality rates from acute myocardial infarction (AMI) per 100,000 population, with corresponding confidence intervals, stratified by gender and by diagnostic support for fatal AMI, for each official Danish region (N = 36,669) between 2002 and 2015. (TIFF) [file pone.0236322.s001.tiff]
